# Study of the Effect of Mint Oil on Nausea and Vomiting During Pregnancy

**DOI:** 10.5812/ircmj.3477

**Published:** 2012-11-15

**Authors:** Hajar Pasha, Fereshteh Behmanesh, Farideh Mohsenzadeh, Mahmood Hajahmadi, Ali Akbar Moghadamnia

**Affiliations:** 1 Fatemeh Zahra Fertility and Infertility Health Research Center, Babol University of Medical Sciences, Babol, Iran; 2 Department of Midwifery, Babol University of Medical Sciences, Babol, Iran; 3 Department of Community Medicine, Babol University of Medical Sciences, Babol, Iran; 4 Department of Pharmacology, Babol University of Medical Sciences, Babol, Iran

**Keywords:** Mentha piperita, Nausea, Vomiting, Aromatherapy

## Abstract

**Background:**

Approximately 80 percent of pregnant women suffer by some degree of nausea and vomiting. But the treatment of nausea and vomiting of pregnancy is rarely successful.

**Objectives:**

The aim of this study was evaluation the effect of mint on nausea and vomiting during pregnancy that its treatment in some recent research has been effective.

**Materials and Methods:**

In this double blind RCT, 60 pregnant women with nausea and vomiting of pregnancy were sampled and divided into two groups with Block-randomized method. mint group, in addition to giving the routine training, for four consecutive nights, before sleeping, a bowel of water whit four drops of pure mint essential oil placed on the floor near their beds and in control groups were used four drops of normal saline . The severity of nausea by using Visual Analog Scale (VAS) and severity of vomiting by counting the number of its in 7 days prior, 4 days during, and 7 days after intervention were assessed.

**Results:**

The results showed that the severity of nausea and vomiting did not differ between the two groups in 7days before and after intervention by using repeated measurement test. But during intervention, the severity of nausea showed a decreasing trend (especially in 4th night) in the mint and an increasing trend in the control group. The severity of nausea within 7 days after the intervention had a decreasing trend in both groups; however, the intensity was lower in the mint than saline group but not statically significant. No meaningful relationship has been detected during and after intervention for the intensity of vomiting.

**Conclusions:**

The results of study showed that peppermint essential oil hasn't the effect on nausea and vomiting of pregnancy.

## 1. Background

Nausea and vomiting are among the common problems in the first half of pregnancy (1). Approximately %80 percent of women are influenced during pregnancy, along with significant impact on their quality of life (2, 3). The reason behind gestational nausea and vomiting is not still well defined (1). In spite of temporal relationship, there is no constant correlation between the severity of nausea and vomiting and increased level of chorionic gonadotropin; however, since conditions with high HCG level, such as molar and multiple pregnancies, are accompanied by higher rates of nausea and vomiting, (4) it seems that nausea is probably caused by increment in estrogen parallel to increase in gonadotropin level (1). Treatment of gestational nausea and vomiting is rarely so successful that the pregnant women could reach to a full recovery. The problem is somehow alleviated by measures such as trying to eat less in more servings, stop eating before satiety, and Nonetheless, vomiting is sometimes so severe that does not respond to treatments; in these cases, drugs such as vitamin B6 (5, 6), promethazine, and are used (1). These drugs are associated with side effects (7). In a study showed that 34% of women did not use drug treatment (vitamin B6), and 26% administered it less than the prescribed dose, and ascribed it to lack of trust in drug safety during pregnancy and the preference to non-medical approach (2). Application of complementary and alternative medicine is the major trend recently occurred in medical care that can even reduce plasma level of stress hormones (8). Although healing ingredients of essential oils are broadly used in medicine throughout the world (9), administration of herbal medicines is limited during pregnancy due to unawareness of their mechanisms of action and lack of randomized controlled trials in this field. Yet, the study showed that 85% of midwives recommend herbal remedies, regardless of their side effects, to pregnant women for treating gestational nausea and vomiting (10). Among the herbal medicines mentioned in recent researches to treat nausea and vomiting of pregnancy, mint can be enumerated (11, 12).

## 2. Objectives

The present study has been carried out to evaluate the effect of mint oil on nausea and vomiting during pregnancy.

## 3. Materials and Methods

**Figure 1:**
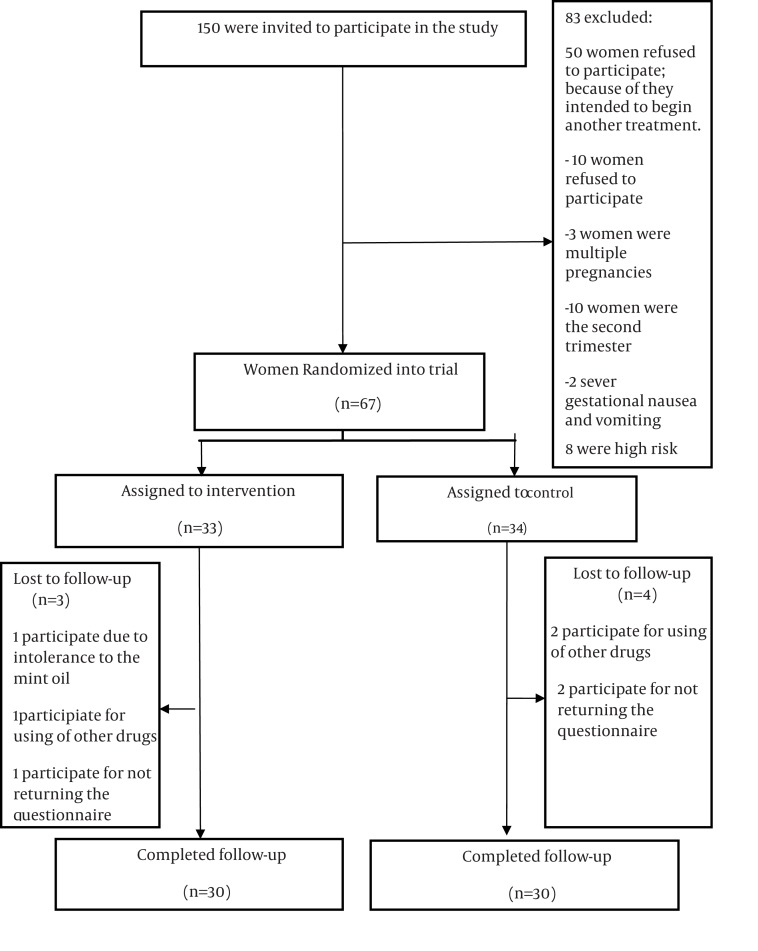
Flow Diagram of Participants Through each Stage of Randomized, Controlled Trial

This double blind clinical trial was conducted, after getting approval by Ethics Committee of Babol University of Medical Sciences and permission for research implementation, on 60 pregnant women complaining of gestational nausea and vomiting sampled by the researcher from prenatal ward of seven selected health clinics based on inclusion (14-35 years, singleton gestation, first trimester pregnancy) and exclusion criteria; well-known underlying physical or psychological problems, dead embryo or fetus with diagnosed malformation, severe gestational nausea and vomiting, multiple gestations and hydatiform mole, and those applied other medication for nausea and vomiting were excluded from this study. Women intended to participate were given the informed consent and were randomly allocated to mint oil (n = 30) and normal saline (n = 30) groups (Figure 1). In addition to receiving the routine training on diminishing gestational nausea, such as more meals and less food per meal, refraining from eating before reaching satiety, avoiding fatty and spicy foods, eating crackers or dry bread before getting up from sleep and keeping hydration (10), the mint group samples were assigned to use a bowl of water with 4 drops of pure mint oil (purchased from Kashan Barij Essence Company) placed on the floor near their beds for four consecutive nights before sleeping to lessen the morning sickness (13, 14). Despite the same instruction to the other group, the placebo samples were given a container with normal saline to use it according to the mentioned approach. It should be noted that both drug and placebo were pre-coded by the consultant pharmacist and were unknown to the researcher and the mother. Some mint oil was poured to inner parts of drug's lid, so that mothers receiving the normal saline cannot be aware of being allocated to this group. The visual analog scale was used to assess the severity of nausea. This objective instrument includes a 10 cm line with areas with a definite beginning and the end and a specified range, on which patients determine their health status. Scores zero and ten are respectively indicative of the best and the worst condition. Nausea intensity-recording visual scale is a self-reporting measure, and since nausea is a sensation felt by patient, it is a highly appropriate technique for measuring the related intensity; in addition, perception and education of recording manner is easy for the study samples (15). To evaluate the severity of vomiting, the frequency of vomiting and retching was counted. Variables such as maternal age, gestational age, education, occupation, place of residence, and BMI were also assessed in terms of group matching. After getting the information, the data were analyzed through descriptive-analytical statistic by SPSS software. Demographic characteristics (i.e., Age, BMI, Gestational age educational level, Occupation, Place of residence) were summarized to characterize the study population. Statistical analyses were performed using t-test (i.e., mean of age, gestational age, BMI), Chi Square (i.e., educational level, Occupation, Place of residence), and repeated measurement (i.e., the severity of nausea before, during, and after intervention in the study groups, the severity of vomiting before, during, and after intervention in the study groups) to determine potentially significant associations, and a p value less than 0.05 was considered significant.

## 4. Results

The results showed similarity between the two groups regarding the maternal age, gestational age, education, occupation, place of residence, and BMI. The minimum and the maximum ages were respectively 14 and 34 years old, 45% of participants were in the age range of 20-25 years. Most of the samples (25%) were in their 8 weeks of gestation (Table 1).

**Table 1:** Comparison of demographic characteristics between two groups of mint oil and normal saline

	Mint oil	Normal saline	P value
**Age, Mean ± SD**	24.8 ± 3.56	25.1 ± 4.76	0.783
**BMI, Mean ± SD**	24.84 ± 2.99	25.54 ± 3.81	0.434
**Gestational age, Mean ± SD**	9.07 ± 1.31	9.73 ± 2.21	0.161
**Education, No (%)**			0.530
< High school	9 (30)	10 (33.3)	
High school	18 (60)	14 (46.7)	
University	30 (10)	6 (20)	
**Occupation, No (%)**			0.646
Housekeeper	26 (86.7)	26 (86.7)	
Employed	4 (13.3)	4 (13.3)	
**Place of residence, No (%)**			0.602
Rental	15 (50)	15 (50)	
Personal	15 (50)	15 (50)	

Results showed similar intensity of nausea and vomiting from 7 days before the intervention. The mean of nausea and vomiting intensity was 4.78 ± 1.62, 4.85 ± 1.82 and 3.00 ± 2.19, 2.52 ± 2.4 in mint and saline groups (P value = 0.865, 0.389).

**Figure 2:**
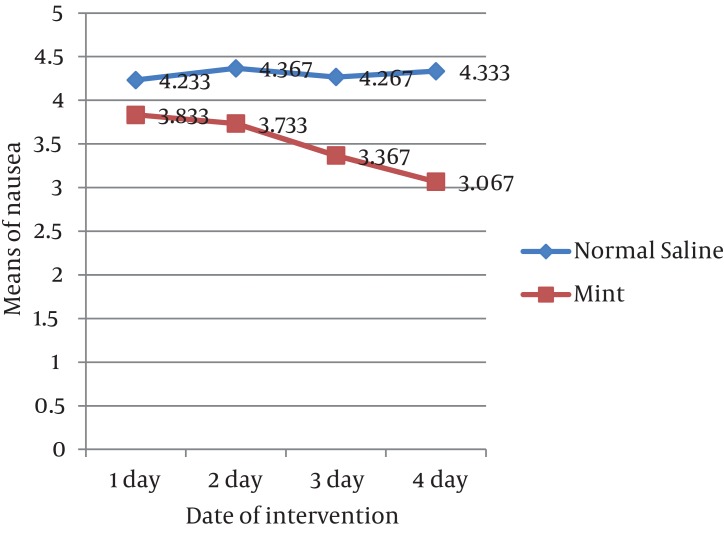
The severity of nausea during the 4 days of intervention in normal saline and mint groups (4.30 ± 2.39, 3.59 ± 2.52)

In the first to fourth days of intervention, the severity of nausea showed a decreasing trend (especially in the fourth night) in the mint and an increasing tendency in the control group (Figure 2).The mean of nausea intensity in mint and saline groups was 3.50 ± 1.95, 4.38 ± 2.18 (P value = 0.140).The mean of vomiting intensity within 4 days the intervention in mint and saline groups was 2.23 ± 1.88, 2.55 ± 2.55 (P value = 0.577).

It has also been observed that within 7 days after the intervention, severity of nausea had a decreasing trend in both groups; however, the intensity was lower in the mint than the control but not statistically significant .No meaningful relationship has been detected seven days after the intervention for the intensity of vomiting.

## 5. Discussion

According to findings of the present study, mint oil aromatherapy has not been effective in reducing gestational nausea and vomiting; although it led to decline in nausea intensity during the intervention in the mint group (especially on the fourth night) and after intervention in both groups (more in the mint group), the difference was not statistically meaningful, that it could be probably due to the small sample size used in the study.

Different results have been brought about by studies conducted on the effect of mint on nausea and vomiting. Some studies have suggested that aromatherapy can relieve nausea or vomiting in the first trimester of pregnancy and also during the labor (9, 16, 17). Researches indicate an increasing percentage of mint administration and support it for relieving nausea and vomiting during pregnancy; in other studies, mint has been used to reduce the morning sickness during pregnancy in 41% of cases (18). However, owing to the use of other medications by patients for symptoms alleviation, no precise scientific connection was found between aromatherapy and nausea abatement (11, 19).

Other investigations have presented ineffectiveness of peppermint on gestational nausea and vomiting. In the same study showed that although ginger, mint and cannabis have been beneficial to treat nausea and vomiting caused by other conditions such as chemotherapy and surgery but only ginger was as the anti-nausea drug in pregnancy (20). Similar to the present study, mint has been ineffective on nausea and vomiting during pregnancy in this research. Likewise, Anderson and colleagues (2004) has reported that peppermint oil has been effectively useful to reduce the severity of nausea after the surgery, in which mint was compared with isopropyl alcohol and placebo (saline), positive effects of aromatherapy have been suggested to be mainly associated to the controlled breathing than the aromatherapy itself since reducing effect of saline was similar to that of peppermint and alcohol (21).

On the other hand, Noureddini (2005) demonstrated that oral use of peppermint essential oil contributes to reversible reduction in gastric acid secretion in rats and, therefore, recommended it to patients with gastrointestinal problems (22). The reason behind such a controversy between the mentioned survey and the present research may be for types of the study samples used. Due to differences in the mechanisms existing in human and animals and, more importantly, dependence of nausea on psychological factors and individual condition, the same results cannot be observed in animal studies and human researches. Among the many limitation of the study, participants' different responses to mint oil aromatherapy can be enumerated as it was very pleasant to some and disgusting to others. Basically, in aromatherapy, each patient needs to smell a particular odor based on his/her own social and psychological conditions and reacts to a specific aroma. Not measuring the hormones level plausibly affecting the gestational nausea and vomiting, such as estrogen, progesterone and HCG, was another constraint of the study; albeit, the probable effect of this limitation was tried to be declined through the measurement of nausea and vomiting 7 days prior to the intervention. Considering the decreasing trend of the intensity of nausea during the intervention, and lower rates of nausea 7 days after the intervention in the mint group, more precise findings can be achieved by further investigations and larger sample size.
